# Bilingualism and Aging: Implications for (Delaying) Neurocognitive Decline

**DOI:** 10.3389/fnhum.2022.819105

**Published:** 2022-02-02

**Authors:** Federico Gallo, Vincent DeLuca, Yanina Prystauka, Toms Voits, Jason Rothman, Jubin Abutalebi

**Affiliations:** ^1^Centre for Cognition and Decision Making, Institute for Cognitive Neuroscience, National Research University Higher School of Economics, Moscow, Russia; ^2^Centre for Neurolinguistics and Psycholinguistics (CNPL), Vita-Salute San Raffaele University, Milan, Italy; ^3^PoLaR Lab, AcqVA Aurora Centre, UiT-The Arctic University of Norway, Tromsø, Norway; ^4^Centro de Investigación Nebrija en Cognición (CINC), University Nebrija, Madrid, Spain

**Keywords:** bilingualism, cognitive aging, neurodegenarative diseases, executive functions, cognitive reserve

## Abstract

As a result of advances in healthcare, the worldwide average life expectancy is steadily increasing. However, this positive trend has societal and individual costs, not least because greater life expectancy is linked to higher incidence of age-related diseases, such as dementia. Over the past few decades, research has isolated various protective “healthy lifestyle” factors argued to contribute positively to cognitive aging, e.g., healthy diet, physical exercise and occupational attainment. The present article critically reviews neuroscientific evidence for another such factor, i.e., speaking multiple languages. Moreover, with multiple societal stakeholders in mind, we contextualize and stress the importance of the research program that seeks to uncover and understand potential connections between bilingual language experience and cognitive aging trajectories, inclusive of the socio-economic impact it can have. If on the right track, this is an important line of research because bilingualism has the potential to cross-over socio-economic divides to a degree other healthy lifestyle factors currently do not and likely cannot.

## Introduction

Cognitive aging refers to the physiological processes of decline in cognition and brain functioning as age increases. Behaviorally, non-pathological cognitive aging is associated with a decrease in overall processing speed, certain types of memory (e.g., short term and episodic), language, visuospatial and executive control. Cognitive aging is marked by significant individual variation in cognitive performance. At the neural level, cognitive aging is perhaps most clearly identifiable in anatomical changes in gray and white matter integrity, particularly in the prefrontal cortex and hippocampus, paired with decreased efficiency (i.e., increased recruitment of implicated networks) in task performance (e.g., Persson et al., 2006; Dennis and Cabeza, 2008; Giorgio et al., 2010; Farokhian et al., 2017; MacPherson and Cox, 2017). Healthy cognitive aging and age-related neuropathologies, such as mild cognitive impairment (MCI) and dementia, can be assessed at the level of functional neural connectivity, the integrated relationship between separated brain regions, as measured by various neuroimaging methods such as functional magnetic resonance imaging (fMRI; Rogers et al., 2007) and resting-state electroencephalography (RS-EEG; Fleck et al., 2017; Babiloni et al., 2018).

Amassing research shows a significant relationship between increased engagement with various lifestyle choices/factors over the lifespan and protection against typically observed cognitive decline in older age (e.g., Stern, 2003, 2007; Goh and Park, 2009; Vemuri et al., 2014; Dause and Kirby, 2019). At present, it is well-known and largely accepted that physical exercise, healthy nutrition, consistent engagement with various leisure activities and higher levels of education, for instance, correlate with greater resilience to the symptoms of healthy and pathological cognitive aging (e.g., Yaffe et al., 2009; Hötting and Röder, 2013). It has also been observed that significant decreases in cognitively stimulating lifestyle activities, specifically within the latter years, are linked to subsequent degree of decline, for example, in speech rate, episodic and semantic memories (e.g., Small et al., 2012). Given their potential benefits, it is important for research to hone in on a more comprehensive list of lifestyle factors with ameliorative potential for age-related cognitive decline.

In this review, we examine the case of bilingualism (see Gallo et al., 2020 for a review), and, most importantly, the conditions under which bilingualism may be considered a relevant protecting factor. In this regard, bilingualism is not “special,” *per se*, compared to other environmental factors that strain and thus train various neural networks, however, its omnipresence in daily life and throughout the lifespan makes it rather unique. Recent meta-analyses report that active bilingualism is related to later *onset* of symptoms and, thus, diagnosis of dementia by as much as 5–7 years relative to comparable monolinguals, despite brains in both cases accruing increased pathology similarly (Anderson et al., 2020; Brini et al., 2020; Paulavicius et al., 2020). While a longer life of active bilingualism seems to convey the greatest protection, bilingual effects are not solely characteristic of native childhood bilingualism. They also pertain to active adult second language learners (e.g., Kuhl et al., 2016; Nichols and Joanisse, 2016; Pliatsikas et al., 2017; DeLuca et al., 2019a; Gallo et al., 2021). This is not surprising as it has been shown that adult second language acquisition, particularly in contexts of active use and engagement such as in linguistic immersion, is related to changes of brain structure and function (Abutalebi and Green, 2016; Calabria et al., 2018; Fedeli et al., 2021) where language processing and control cross-over with executive functioning (see Pliatsikas, 2019 for review).

However, as outlined below, simply being bi- or multilingual it is not sufficient for protection against cognitive decline, otherwise, a clear majority of the world’s population would be equally protected (considering that more than half of the world population speaks more than one language; De Houwer, 2021). It is important to keep in mind that only certain types of so-called ′active ′ bilingualism will have the maximum effect upon brain health, i.e., those who are amply exposed to their languages, use them regularly and are otherwise highly engaged in contexts that require linguistic switching. Before providing evidence speaking to this, we should address an obvious question: what is the cognitive mechanism that bilingualism engages rendering the brain more resilient against decline?

**FIGURE 1:**
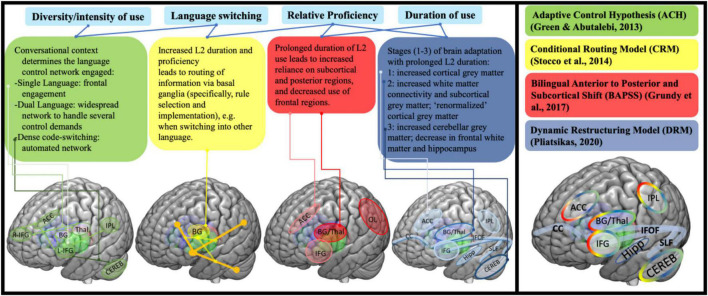
Overview of selected models of bilingualism and neural adaptations based on various aspects of bilingual experience (Green and Abutalebi, 2013; Stocco et al., 2014; Grundy et al., 2017; Pliatsikas, 2020). Overlap in key brain areas/structures implicated in bilingualism depicted in the summary panel on the right.

Bilingual speakers are continuously faced with an extra burden on cognitive functioning: unlike monolinguals, they have to control their languages. Research shows that all the languages of bi-/multilinguals are constantly activated regardless of contextual need or conscious intent (Spivey and Marian, 1999; Jared and Kroll, 2001; Kroll et al., 2008; Green and Abutalebi, 2013). The languages must be managed for shifting selection of the one matching any given communicative context (Green and Abutalebi, 2013), thus, avoiding unwanted interference from the contextually irrelevant one. For bilinguals, the need to switch from one language to the other in the real world is dynamic, complex and not always predictable. After all, one could be using language X or be codeswitching between languages X and Y (because the interlocutor context allows and calls for this) when someone associated strictly with language Y enters the room or calls. Unexpected as it might be, instantaneous switching to a (different) unilingual mode becomes required. This constant suppression of the contextually irrelevant language via substantial and continuous recruitment of executive control processes is argued to “train” related cognitive skills and brain networks, making them more efficient. Indeed, this “training” has been shown to change domain-general processes and the underlying neural architecture directly and fundamentally (e.g., Bialystok et al., 2008; Baum and Titone, 2014; DeLuca et al., 2019a). Indeed, in recent years, several—not mutually exclusive—models have schematized how the brain structurally and functionally accommodates the dynamics of the implicated competition-based management (e.g., intensity and duration of language use and switching behavior), for example, those in Figure 1 (adapted from DeLuca et al., 2020) where the combined image on the right shows the models’ convergences and distinctions.

Interestingly, such unwanted intrusions from the contextually irrelevant language are common in the case of brain pathology where it is argued that the so-called language control network is knocked out, such as in bilingual aphasia and certain types of dementia involving the frontal lobes (Abutalebi and Green, 2016).

In recent years, mixed reporting, especially in the behavioral bilingual executive control literature, have fostered debate over the extent to which bilingualism actually results in demonstrable gains in relevant performance (e.g., Duñabeitia et al., 2014; Paap et al., 2015; Nichols et al., 2020; see Lehtonen et al., 2018 for meta-analysis). An important issue of comparability across studies complicates any generalizable or definitive conclusions on the matter, essentially the lack of a universal way to qualify and quantify degree of bilingualism across studies (see Leivada et al., 2021 for discussion). Contemporary research treating bilingualism as a complex spectrum of experiences has shown that certain levels of activity and engagement with bilingualism are linked with increased probabilities for measurable knock-on effects (e.g., Kuhl et al., 2016; Gullifer et al., 2018; DeLuca et al., 2019b; Luo et al., 2019; Gallo et al., 2021). It seems that bilingualism *par excellence*, as aforementioned, is not a sufficient qualifier, but rather active engagement with bilingual experiences provides increased opportunity for mental conflict and resolution to drive eventual adaptation. Thus, measures of dichotomous categorization between bilinguals and monolinguals (“yes” or “no”) are simply inadequate.

Beyond behavioral cognitive function measures, there is greater consistency of findings across bilingual neuroimaging studies at all ages. As we review in detail below, a plurality of relevant studies shows changes in the bilingual brain that overlap topographically with regions highly implicated in language processing/control, memory, and executive functioning (e.g., De Baene et al., 2015; Abutalebi and Green, 2016; Calabria et al., 2018). fMRI studies show increased efficiency in neural recruitment during task performance in bilinguals, even when there are no measurable behavioral differences (e.g., Abutalebi et al., 2012; DeLuca et al., 2020). Neuroimaging is not dependent on behavioral effects alone, a significant asset since there are non-trivial issues with granularity and test-retest reliability for many of the cognitive behavioral tasks in common use. More recently, neuroimaging work measuring bilingualism as relative engagement also demonstrates how more active bilingualism (increased exposure, domains of use, proxies for social use and networking, etc.) is linked with individual-level neuroanatomical change/more efficient neuronal recruitment during behavioral task performance (Dash et al., 2019; DeLuca et al., 2019b; Sulpizio et al., 2020).

Given the aims and discussions we have herein, it is crucial to point out that the functions and brain regions argued to be enhanced by bilingualism largely overlap with those that decline due to cognitive aging, MCI, and dementia. As we will see below, research comparing older bilinguals to monolinguals in terms of cognition shows that bilinguals outperform monolinguals in non-linguistic tasks tapping executive control processes (e.g., Bialystok et al., 2004; Luo et al., 2013). As highlighted by Valian (2015), the effects of bilingualism upon these cognitive processes are more evident in elderly populations because typically these individuals are less exposed to other cognitively stimulating tasks such as musical activities, intellectual exercise, videogaming and social media. For the very same reason, the effects of bilingualism may be masked by those additional cognitively challenging activities in younger subjects, which may partially explain the discrepancy in the behavioral literature comparing younger bilinguals to their monolingual peers (see above).

In terms of brain function, older age bilinguals demonstrate lower activation of regions supporting executive control tasks (Gold et al., 2013b), and increased resting state connectivity within related networks (Pliatsikas and Luk, 2016), suggesting increased neural efficiency. In terms of brain structure, they show better preserved gray matter in regions related to executive control, and better-preserved white matter in the tracts that connect them (Abutalebi et al., 2014; Pliatsikas, 2020). Moreover, while healthy monolingual seniors show a decoupling of age with brain structure, this is not the case for bilinguals (Anderson et al., 2021). Taken together, such evidence strongly suggests bilingualism can impinge on timing and degree of cognitive and/or neural decline. In what follows, we review this literature in greater detail before offering some discussion to be considered with regard to how this evidence might, on multiple levels, lead researchers and societal stakeholders to act in the near future.

## Effects of Bilingualism on Pathological Aging

The first reported evidence of bilingualism-induced effects during pathological aging came from retrospective cross-sectional studies, comparing bilingual and monolingual older adults on their clinical history, disease severity and symptoms onset. A seminal study in this regard was that of Bialystok et al. (2007), in which timing in terms of age of disease onset of Alzheimer’s Disease (AD) was compared between bilingual and monolingual samples matched for gender ratio, maximal educational attainment and socioeconomic status, namely factors that are known to affect the dementia trajectory (Meng and D’Arcy, 2012; Sattler et al., 2012). Bialystok et al. (2007) found that, on average, bilinguals presented with symptoms of dementia 4 years later than monolinguals. Such a prominent difference raised attention and led to numerous attempts to replicate these findings. Despite a subsequent study by the same group reporting consistent evidence (Craik et al., 2010), two further investigations early on suggested that bilingualism effects against pathological aging may be restricted to certain social instances. A study by Gollan et al. (2011) found a relationship between level of bilingualism and delay in AD onset in low-educated, but not in highly educated Spanish-English bilingual US residents. Similarly, a study by Chertkow et al. (2010) found evidence of a bilingualism-related delay of AD only in immigrant, but not in non-immigrant bilinguals.

Subsequent evidence, however, provided findings that seemed to contradict these studies. As per the education factor, a study by Ramakrishnan et al. (2017) compared the contribution of education and bilingualism in delaying MCI in a sample of 115 individuals (more than twice the sample size of Gollan et al. (2011) study, which included 44 participants). They found that while bilingualism delayed MCI onset by 7.4 years, education had no beneficial effect in their sample. Regarding immigrant status, the argument that any effect might be reducible to the challenges—cognitive, linguistic and otherwise—related to immigrating to another country over any separable role of bilingualism *per se* is appealing. Moreover, a series of confounds related to a potential imbalance of other environmental factors also associated with healthy aging may be intrinsic to comparing immigrant and non-immigrant samples, for example, possible differences in dietary and health habits, education, and cultural background. In the aforementioned studies, these factors were unfortunately not controlled for across aggregates. Evidence from Alladi et al. (2013) has, however, proven crucial to this debate. In a large population study including data from 648 senior AD patients from India, all non-immigrants, AD age of onset was compared between monolingual and bilingual groups. The results confirmed a 4.5-year delay in symptom onset for non-immigrant bilinguals as compared to monolingual peers. The study also provided further evidence regarding the role of education: while the delay was even larger (i.e., 6 years) in low-educated bilinguals, the effect persisted in highly educated bilinguals as well. Early results were replicated in several other subsequent investigations: a 2014 study by Bialystok et al. (2014a) investigated disease onset in 74 MCI and 75 AD monolingual and bilingual patients, revealing a delay of 4.7 and 7.3 years, respectively, in the bilingual sample. Importantly, in these later studies where other cognitive aging ameliorating variables were considered, this result emerged irrespectively of lifestyle variables such as dietary and smoking habits, level of alcohol consumption and amount of physical activity or social interactions. Moreover, a study by Woumans et al. (2015) reported a 4.6-year delay in AD onset in bilinguals compared to a group of monolinguals matched for gender ratio, maximal educational attainment, occupational complexity, and severity of cognitive impairment. Other recent retrospective studies provide corroborative evidence: for instance, Zheng et al. (2018) found a 7-year delay of AD onset in Cantonese/Mandarin bilingual older adults as compared to Cantonese and Mandarin monolingual peers. Finally, a study by Mendez et al. (2019) replicated the finding of a 4-year AD delay in a sample of bilingual seniors who spoke various first languages (L1s) and English as a second language (L2).

Recent meta-analyses confirm an overall effect of bilingualism in delaying AD’s symptoms onset. Anderson et al. (2020) reported a moderate effect size evidence for a delaying effect of bilingualism on AD (Cohen’s *d* = 0.4), although only weak evidence for protection from contracting AD altogether (Cohen’s *d* = 0.1, but see below for further discussion on the relationship between bilingualism and AD incidence). Importantly, these results controlled for the influence of socioeconomic status, maximal educational attainment as well as publication bias. In another meta-analysis including data from 8 studies, Paulavicius et al. (2020) found that, on average, bilinguals exhibited AD symptoms 4 years later and were diagnosed with AD 2 years later relative to monolinguals. Finally, a meta-analysis of 21 studies (Brini et al., 2020) reported that bilinguals experienced AD 4.7 years and received AD diagnosis 3.3 years later than monolinguals but found no significant difference in dementia incidence between the two groups.

Some studies have also investigated the effect of bilingualism on different sub-types of AD and MCI, providing an even more detailed account of the consequences of bilingualism for pathological aging. For example, a study by Ossher et al. (2013) reported a 4.5-year delay in the onset of amnestic MCI (aMCI; the kind of MCI specifically characterized by memory dysfunction) for bilinguals as compared to monolinguals, but only in patients suffering from single—and not multiple—domain aMCI (with the difference between the two being the number of cognitive domains impaired; Winblad et al., 2004). Importantly, single-domain aMCI is the aMCI type most likely to convert to full-blown AD. A more recent study by de Leon et al. (2020) found a 5-year delay in AD onset in bilinguals suffering from the logopenic variant of primary progressive aphasia (a neurodegenerative variant of aphasia characterized by difficulties with finding words and reduced output but relatively preserved syntax and phonology; Henry and Gorno-Tempini, 2010), but not in those suffering from the amnestic variant of AD, relative to monolinguals.

Moreover, potential effects of bilingualism are argued to, in principle, extend beyond AD, dementia and MCI. In other words, it is at least theoretically reasonable, and there is some emerging evidence to suggest, that bilingual engagement could be a protective factor in clinical and pathological aging more generally, extending to other specific types of neurodegeneration. The rationale behind this, as laid out in Voits et al. (2020), lies in the fact that there is also an overlap between brain areas affected by neurodegenerative diseases such as Huntington’s disease, Parkinson’s disease, multiple sclerosis and those implicated in bilingual language processing and control. Intuitively, this suggests the potential link between them: bilingualism would “train” and reenforce brain areas and their respective connections that are susceptible to decline in the face of multiple types of neuropathology. Testing this assumption is a reasonable path of research that is likely to become more prevalent in the near future given the putative role of bilingualism as a cognitive and brain reserve enhancer (Gallo et al., 2020; Bialystok, 2021; see also section “Underlying Mechanisms and Models of Bilingualism-Induced Successful Aging” below for further discussion). Indeed, higher levels of brain reserve have been shown to lead to better outcomes in a range of neurodegenerative diseases (Sumowski et al., 2009; Hindle et al., 2017). And yet, there is a scarcity of empirical research directly examining potential links between bilingualism and non-AD neuropathology.

Although more research is required, results from the small body of existent literature are encouraging. Bilingual Huntington’s disease patients have been reported to have increased metabolism across multiple brain areas as well as structurally increased GMVs in the inferior frontal gyrus, resulting in increased cognitive functioning (Martínez-Horta et al., 2018). Bilingualism has also been shown to lead to later onset of Parkinson’s disease symptoms (Ghazi-Saidi, 2019), although there is no evidence for better cognitive functioning in bilingual patients in the two studies on cognitive outcomes published to date (Hindle et al., 2015; Fishman et al., 2021). There is also some evidence pointing toward improved cognitive outcomes in multiple sclerosis patients (Soltani et al., 2018; Aveledo et al., 2020). Finally, although stroke is sudden and has an acute onset, rather than progressive neurodegeneration, bilingualism has been linked to improved post-stroke cognitive outcomes (Alladi et al., 2016), and less severe symptoms of post-stroke aphasia (Paplikar et al., 2018). Note, however, that these studies examine behavioral outcomes only. A neuroimaging component is warranted, if not needed, to fully understand bilingualism-induced reserve effects and putative compensatory effects on an array of neurodegenerative disorders.

While the above literature convincingly establishes a delaying effect linked with bilingualism, a parallel literature has investigated potential differences in incidence of MCI and AD in bilingual and monolingual populations. Perquin et al. (2013), for example, retrospectively investigated MCI incidence in a sample of 232 participants, revealing an inverse relationship with the number of languages spoken by individuals. In a subsequent prospective (or longitudinal) study, Wilson et al. (2015) followed a cohort of 964 seniors over a 6-year span. MCI incidence was significantly lower in the bilingual population. Finally, Klein et al. (2016) proposed a more global approach: they investigated the relationship between the degree of multilingualism and dementia incidence in 93 countries, revealing a bilingualism-induced protective effect against the development of AD diffused worldwide. This result is particularly important with reference to the debate on the sociocultural boundaries of potential bilingualism-induced benefits on pathological aging.

Yet another parallel stream of research has investigated the consequences of bilingualism on pathological aging with neuroimaging techniques. The first study of this kind, Schweizer et al. (2012) investigated differences in bilingual and monolingual individuals suffering from AD, matched for maximal educational attainment and disease severity, using computerized tomography. For comparable levels of symptoms severity, bilinguals exhibited more severe brain atrophy in regions of the medial temporal lobe, an area that plays a crucial role in declarative memory and is targeted by AD in very early stages of the disease (Frisoni et al., 2002; Zhang et al., 2008). At first glance, this result might appear as contradictory to any claims of a beneficial role of bilingualism. However, considering that bilinguals and monolinguals were matched for symptom severity, the interpretation of the findings is rather advantageous: bilinguals are able to compensate for neural damage to a larger extent than monolinguals, i.e., to maintain similar cognitive efficiency in the face of more severe AD-induced brain deterioration. In line with these results, in an investigation with fluorodeoxyglucose positron emission tomography (FDG PET), Kowoll et al. (2016) reported reduced glucose uptake (a biomarker associated with AD) in frontotemporal, parietal and cerebellar regions of bilinguals diagnosed with MCI and probable AD, relative to a sample of monolinguals matched for age, gender ratio and disease severity. Similar results were obtained in two subsequent FDG PET investigations (Perani et al., 2017; Sala et al., 2021) where AD bilingual patients were found to have more severe glucose hypometabolism, as well as increased functional connectivity in the executive and default mode network (we unpack the significance of increased functional connectivity in the next section), as compared to monolinguals matched for disease duration and overall cognitive efficiency. Strikingly, the average age of the bilingual AD cohort was 4.5 years older than the monolingual AD group, replicating the findings of the abovementioned clinical studies. Another study (Estanga et al., 2017) reported a mitigating effect of bilingualism on the amounts of t-tau protein, a cererebrospinal fluid (CSF) biomarker of AD, in a sample of 278 participants with comparable genetic, socio-demographic and cognitive profiles. Finally, a combined cross-sectional/longitudinal investigation (Costumero et al., 2020) revealed that bilingual MCI patients had significantly lower raw amounts of brain parenchyma (i.e., gray + white matter) as compared to monolingual peers matched for general cognitive functioning. The longitudinal stage of the study also showed more severe parenchyma loss and cognitive decline in monolinguals in the average follow-up time of 7 months.

## What Effects Does Bilingualism Confer on Healthy Aging?

The question of whether bilingual effects in older age are only (or perhaps best) attested in pathological neurodegeneration is an interesting one. In principle, the answer should be “no,” although they might be more challenging to examine in healthy populations. Due to the involvement of executive control in language control processes and its potential as a general “trainer” of the mind and brain together with the mere length of bilingual experience in elderly individuals, it is reasonable to expect effects for senior bilinguals, particularly in tasks that tap into relevant cognitive ability, regardless of (a) typicality of cognitive aging. Indeed, several studies report evidence that bilingualism is linked to increased preservation of executive functioning in otherwise healthy older adults. More specifically, senior bilinguals have been repeatedly shown to outperform monolingual peers on executive control tasks that include, but are not limited to, the Simon Task, the Flanker Task and the Stroop Task (Bialystok et al., 2004, 2008, 2014b; Gold et al., 2013b; Abutalebi et al., 2015b; Estanga et al., 2017; Del Maschio et al., 2018; Incera and McLennan, 2018; López Zunini et al., 2019). Bilingualism-induced effects on healthy aging, perhaps even more interestingly, seem to extend beyond executive control: they have also been reported for executive-related memory recall tasks (Wodniecka et al., 2010; Ljungberg et al., 2013), working memory (Bialystok et al., 2014b), semantic memory (Arce Rentería et al., 2019) and even general intelligence (Bak et al., 2014).

**FIGURE 2:**
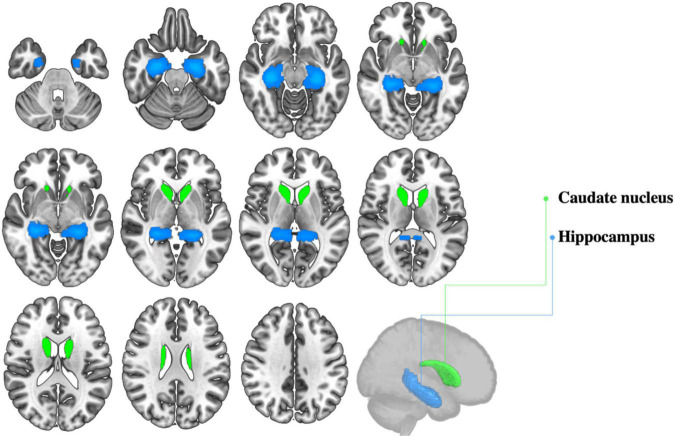
Selected subcortical gray matter structures shown to be sensitive to bilingual experience.

Evidence that bilingualism may play a protective role against non-pathological age-related neurocognitive decline is not limited to behavioral investigations: several neuroimaging studies suggest that bilingualism can foster the preservation of neural structure and function in healthy aging. One consequence, for example, is enhanced preservation of gray matter integrity in several brain regions. These regions, mainly part of the language control network (Abutalebi and Green, 2016), include the temporal pole (Abutalebi et al., 2014; Olsen et al., 2015), the orbitofrontal cortex (Abutalebi et al., 2014), the inferior parietal lobule (Abutalebi et al., 2015a; Del Maschio et al., 2018), the anterior cingulate cortex (Abutalebi et al., 2015b; Del Maschio et al., 2018), the prefrontal cortex (Del Maschio et al., 2018), and the hippocampus (Voits, 2020; Voits et al., 2022). Importantly, some of these areas are particularly sensitive to early stages of age-related brain deterioration, as in the case of the temporal pole (Kalpouzos et al., 2009), the inferior parietal lobule (Apostolova et al., 2007) and the hippocampus (Maruszak and Thuret, 2014). In this light, studies showing enhanced preservation of gray matter density in such areas have particular relevance (see Figure 2 for sample subcortical regions that have been shown to be sensitive to bilingual experience). For instance, Abutalebi et al. (2014) reported higher GMVs in the temporal pole of healthy senior bilinguals relative to monolingual peers. Moreover, bilinguals’ temporal pole GMVs showed a positive association with proficiency in the L2, indicating a dose-response effect of bilingualism on neuroplasticity in this area. Similarly, Olsen et al. (2015) found greater cortical thickness in the temporal pole of healthy bilingual older adults, as compared to age-matched monolinguals. As per the inferior parietal lobule, available evidence shows bilingualism-induced neuroplastic changes that are not limited to late life stages (Abutalebi et al., 2015a; Del Maschio et al., 2018), but extend to young adulthood (Mechelli et al., 2004; Del Maschio et al., 2018) and even childhood (Della Rosa et al., 2013), suggesting that consequences of bilingualism for cognitive aging may be rooted in early life stages.

**FIGURE 3:**
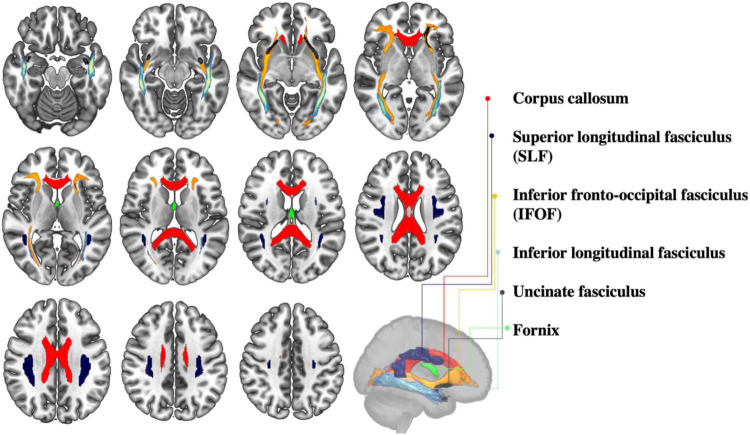
Selected white matter tracts shown to be sensitive to bilingual experience.

In addition to gray matter, bilingualism has also been shown to be linked with white matter preservation during the aging trajectory (Figure 3). Using diffusion tensor imaging (DTI), Luk et al. (2011) reported higher levels of white matter integrity for healthy bilingual seniors, relative to monolinguals, in the corpus callosum, the superior longitudinal fasciculi, the right inferior fronto-occipital fasciculus and the right uncinate fasciculus, namely tracts that are particularly targeted by age-related deterioration (Gunning-Dixon et al., 2009). In the same study cited above, Olsen et al. (2015) found higher white matter volumes in the frontal lobe of their bilingual sample, compared to the monolinguals. Finally, in another DTI investigation, Anderson et al. (2018) found that bilingualism was linked to white matter integrity preservation in the left superior longitudinal fasciculus.

Thus far, we have discussed bilingualism-related effects on the aging brain at the structural level, but supporting evidence also extends to the functional level. Indeed, bilingualism has been shown to also positively affect functional neural mechanisms during senescence. For instance, in an fMRI investigation, Gold et al. (2013b) reported lower switching costs, i.e., the contrast between the level of activation of brain areas during trials that entail switching vs. trials that do not—for healthy bilingual older adults, relative to monolinguals, in areas of the language control network: namely, the left dorsolateral and ventrolateral prefrontal cortices and the anterior cingulate cortex. In addition, the task performance of bilingual seniors was found to resemble those of younger participants of the study, in that both groups outperformed monolingual seniors while showing reduced activation in prefrontal regions. Ansaldo et al. (2015) showed differential recruitment between healthy bilingual and monolingual seniors during a Simon Task. In incongruent trials (i.e., those requiring executive control), despite performing behaviorally at comparable levels, monolinguals activated the right middle frontal gyrus, while bilinguals relied on the inferior parietal lobule. Thus, bilinguals appeared to avoid the typical tendency to shift task-related activation to more anterior regions during aging, which is thought to depend on age-related depletion of posterior-parietal neural resources (Davis et al., 2008, see section “Underlying Mechanisms and Models of Bilingualism-Induced Successful Aging” below for further discussion). Dash et al. (2019) examined brain activation using fMRI during the Attention Network task. They tested older bilinguals with varying degrees of proficiency in their L2 and found a relationship between neural efficiency (as indexed by decrease in neural activity) and L2 proficiency, thus suggesting that the effects of bilingualism are not monolithic in older ages but rather vary as a function of L2 experiential factors, as shown in younger populations as well (e.g., Li et al., 2014; DeLuca et al., 2019b; Gallo et al., 2021).

Available evidence also supports a protective role of bilingualism against age-related disruption of functional connections in the brain. For instance, an fMRI study mentioned above (Luk et al., 2011) revealed better preservation of anterior-to-posterior resting-state functional connectivity (rs-FC) in elderly bilinguals, compared to age-matched monolinguals. Similar results have been reported regarding rs-FC within the default mode- and executive control networks (Grady et al., 2015). Corroborating evidence comes from electrophysiological investigations as well: a magnetoencephalography (MEG) study by de Frutos-Lucas et al. (2020) highlighted better-preserved rs-FC for healthy bilingual seniors, compared to monolingual peers, in five occipito-parietal clusters particularly sensitive to AD-related disruption (Nakamura et al., 2017). Finally, in a study investigating task-related functional connectivity during a Simon Task, Berroir et al. (2017) reported that healthy bilingual seniors needed to allocate fewer resources to perform the task, showing increased connectivity only in visuospatial areas (i.e., the inferior temporal sulcus), compared to monolinguals who showed greater connectivity in a set of areas linked to visual, motor, executive and interference control processing.

In terms of neuroimaging evidence, perhaps the most interesting bilingualism-induced effect on healthy cognitive aging is one having to do with modulation. In a handful of studies to date, bilingual experience appears to mitigate one of the most well-known relationships in neuroscience, the structural brain-behavior (SBB) one. In general terms, this relationship predicts that for higher density (or integrity) of brain structure in a particular region or network, the relative level of cognitive performance will increase. Conversely, when brain structure deteriorates, performance is expected to worsen. In these regards, Gold et al. (2013a) reported that, for comparable levels of cognitive functioning and matched demographics, bilinguals showed lower white matter integrity than monolinguals in the inferior longitudinal fasciculus, the inferior fronto-occipital fasciculus, the fornix, and parts of the corpus callosum. Since the SBB relationship would have predicted greater cognitive impairment in the bilingual sample, these findings suggest a mitigating role of bilingualism on such relationship. Similar results were obtained in a recent investigation taking the reverse approach: matching bilingual and monolingual healthy seniors on white matter integrity measures, sex, age, and maximal educational attainment, Berkes et al. (2021) examined differences in task performance. Here, bilinguals were found to display higher cognitive functioning than monolingual peers despite their brains being matched, adding to the existing body of evidence of bilingualism’s mitigating effects on the SBB relationship. Another study deploying a Flanker Task and structural MRI (Abutalebi et al., 2015b) revealed that the cost of the conflict effect (i.e., the difference between reaction times in the congruent vs. incongruent trials) was positively correlated with dorsolateral prefrontal cortex GMV for healthy senior monolinguals, but not for bilingual peers. This result was replicated and expanded in a subsequent study by Del Maschio et al. (2018) who found that bilingualism mitigated the SBB relationship in a number of regions of the language control network, namely the bilateral prefrontal cortex, the bilateral inferior parietal lobule, the left anterior cingulate cortex and the left caudate nucleus. These findings suggest that bilingualism may grant protection from age-related cognitive impairment by enabling the maintenance of good cognitive functioning, even in the face of measurable age-related neural deterioration.

## Underlying Mechanisms and Models of Bilingualism-Induced Successful Aging

As reviewed above, sufficient available evidence points to a role of bilingualism as a life experience in fostering successful aging. However, the precise mechanisms underlying such a phenomenon are yet to be clearly illustrated. Here below, we review the main models of bilingualism-induced benefits for cognitive aging, which attempt to understand what lies beneath the relationships observed in empirical studies.

The main theoretical framework adopted to model bilingualism-induced successful aging is that of *reserve*. The theoretical construct of reserve is defined as *the discrepancy between the observed and expected level of cognitive impairment, given the relative degree of age-related neural deterioration* (see Stern, 2009; Stern et al., 2020). To the extent accrued (and maintained) over the lifespan, *reserve* should act as a buffer against age-related decline by providing individuals the necessary hardware (brain) and software (cognition) to *stave off* and *compensate for* cognitive impairment occurring as a function of increasing age and/or neurodegenerative pathology (Steffener et al., 2011; Stern et al., 2020). Reserve has two main sub-components: *brain reserve* and *cognitive reserve*. Brain reserve (BR) acts through the preservation of the neural substrate at the structural level: it is, thus, a *passive* mechanism. Brain reserve would manifest via well-known neuroplastic mechanisms such as neurogenesis (formation of new neurons), synaptogenesis (formations of new synaptic contacts) and angiogenesis (formation of new blood vessels) (Stern, 2009). Cognitive reserve (CR), instead, acts at the functional level by enhancing the efficiency, capacity, and flexibility of neural networks. CR *actively* impacts the neural substrate by providing extra resources to be deployed for high levels of task complexity (i.e., capacity), enabling the optimization of cognitive performance despite lower necessary brain activation (i.e., efficiency) and allowing the use of alternative neural pathways for task resolution (i.e., flexibility) (Stern, 2009).

Crucially to the discussions herein, reserve is argued to originate from environmental influences, such as regular lifestyle activities that exert a “training” effect on the mind and brain. The evidence detailed in the above two sections have all the hallmarks of measurable side-effects expected from CR and BR accrual. As such, to the extent that bilingualism has been isolated as the key variable in specific studies making this claim, the existent evidence base fits well within the reserve framework. For instance, the findings reviewed in the above sections regarding bilingualism linking to structural and functional neuroplasticity align nicely with the concepts of BR and CR. Moreover, the mitigating effect exerted by bilingualism on the SBB relationship appears to adhere well to the general definition of reserve provided above.

The logical question becomes, how do exponents of bilingualism result in both functional and structural adaptations in the brain, meaning how do they translate to BR and CR accrual? The main linking hypothesis, also espoused in our introduction, is the claim that increased executive control effort is required for the simultaneous management of two linguistic systems. As one would expect, not differentially to other lifestyle contributors to reserve, given the nature of bilingualism individual variation in relevant outcomes should be predictable, at least in part,^1^ on the basis of degree of engagement with the underlying mechanism: for the present purposes, the qualitative and quantitative nature of opportunities one has had within their bilingualism. This accords well with the progression of the field and, indeed, the fact that neurocognitive effects of bilingualism are not always attested experimentally; the most contemporary approaches to bilingualism and neurocognition highlight the spectral nature of what it is to be bilingual and the determinism of individual-level engagement with the exponents of the bilingual experience (e.g., Luk and Bialystok, 2013; Li et al., 2014; DeLuca et al., 2019b; Pliatsikas et al., 2020; Gallo et al., 2021).

Several mechanisms and models have been proposed to capture the mapping of bilingual engagement to outcomes of varying types of reserve. Guzmán-Vélez and Tranel (2015) argue that a possible underlying mechanism of bilingualism-induced brain reserve might be found in consequences of bilingualism for the noradrenergic system (a system responsible for the generation and release of the neurotransmitter norepinephrine). The noradrenergic theory of reserve (Robertson, 2013) claims that brain and cognitive reserve would stem from the upregulation of noradrenergic circuits. This is predicted to result in neuroplastic mechanisms that include neurogenesis, angiogenesis, synaptogenesis or heightened production of a brain-derived neurotrophic factor. It also predicts compensatory mechanisms to mitigate the impact of age-related brain pathology, such as reduced amyloid burden, anti-inflammatory processes and recovery of cholinergic and dopaminergic cells. Under such a framework, bilingualism would not be different from any other reserve-promoting factor, acting in the ways just described to enable resilience to age-related cognitive impairment.

Other mechanisms with a more macroscopic focus have also been proposed. These models converge on the notion of dynamic recruitment of different structures and networks to handle existing cognitive demands more efficiently, such as bilingual language control (see Hernandez et al., 2019 for discussion) which in turn are argued to provide a neural basis for reserve (as discussed above). Grant et al. (2014) explain bilingualism-induced promotion of successful aging in terms of the *posterior-to-anterior shift in aging* (PASA; Davis et al., 2008). The PASA model predicts that, due to processing deficits in occipitotemporal regions related to aging, seniors would rely on the compensatory activation of domain-general frontal areas to preserve optimal cognitive performance. However, since bilinguals have been shown to maintain higher integrity of posterior-parietal brain structures such as the inferior parietal lobule or the temporal pole, as well as optimal connectivity within and to the posterior areas during senescence (see section “What Effects Does Bilingualism Confer on Healthy Aging?” above), they would be able to postpone the PASA shifting, saving the deployment of extra neural and cognitive resources for higher levels of task complexity. Building on—and extending—this framework, Grundy et al. (2017) offered the *bilingual anterior to posterior and subcortical shift* (BAPSS) model, which claims that sufficiently engaged bilinguals would actually shift the neural processing related to executive tasks from frontal to posterior and subcortical regions. BAPSS is based on evidence that bilinguals experience neuroplastic changes in posterior and subcortical regions of the brain and increases in white matter integrity, as well as showing decreased task-related frontal activation, increased task-related functional connectivity, and a stronger reliance on earlier processing stages, relative to monolinguals, during executive task performance. Such changes, resulting from bilingual experience, would allow elderly bilinguals to shift to automatic, early, economic executive processing in lieu of effortful, late, top-down mechanisms. As discussed above for the PASA model, delaying the necessity to recruit frontal compensatory regions would allow bilinguals to maintain cognitive resources to be deployed in the event of more demanding task requirements.

The Dynamic Restructuring Model (DRM; Pliatsikas, 2020) makes predictions consistent with the above models, but the specificity of these is more geared toward the structural, as opposed to the functional, trajectory of adaptation. The DRM is based on a model of expansion and renormalization: initial stages of engagement to novel stimuli/cognitive demands confer an increase in regional GMV and white matter structure to handle these. With prolonged experiential engagement, these increases revert back to a state of equilibrium toward “baseline” levels. However, (i) this is not necessarily exactly the same as the original baseline levels and (ii) this return reflects a new arrangement of the most efficient series of synaptic connections with underused connections being pruned (see e.g., Lövdén et al., 2013). Furthermore, this is proposed to occur in a three-stage process. The first (initial exposure) holds greatest cognitive demands with the acquisition of a new skill and is reflected in increased cortical/hippocampal GMVs. In the second state (“consolidation”) these skills are integrated into more of an automated/efficient network; structurally this is reflected as modulations in subcortical gray matter and white matter structure. Finally, a stage of “peak efficiency” or automation in cognitive demands is reached and is reflected in return toward baseline levels for the subcortical gray and frontal white matter structure and increases in cerebellar structure. It should be noted that neurocognitive aging was not in the original scope of the DRM. However, the notion of *renormalization* discussed within it may provide a mechanism by which neural reserve may be observed. If lifelong bilinguals are compared to monolinguals later in life, higher gray- or white matter structure may not reflect “more” gray matter which must decay, but a similar level of overall volume or structure which is more *resilient* to the effects of neural decay (Pliatsikas et al., 2020). Further to this, the shifting reliance toward subcortical, and then posterior regions, with prolonged bilingual exposure could provide a basis for cognitive reserve in line with the predictions from the above models.

## Conclusion

Having reviewed the logic behind and evidence for the potential connection between bilingualism and successful cognitive aging, this section aims to underscore various layers of importance of the research program that endeavors to understand the connections more precisely. Before doing so, it is worth underscoring that while we have focused our review and discussion herein based on brain-level research from MRI studies, combining MRI moving forward with other neuro methods is highly recommended. For example, various types of EEG (ERP and/or oscillatory dynamics) can be insightful, not least as they provide a means to examine task performance in ways MRI cannot. While fMRI can give insights into relevant network recruitment on task, EEG offers much more fine-grained details as it relates to temporal resolution of what the brain is doing in the course of such performances and is perfectly compatible with simultaneous MRI. As we move toward harnessing the insights for application/intervention from the basic science research looking into potential connections between bilingualism and cognitive aging, research that employs other methods, such as Trancranial direct current stimulation (tDCS) and/or Transcranial magnetic Stimulation (TMS), are also welcome. While these methods have both basic science application and therapeutic potential, especially as paired with cognitive training for the functional connectivity of working memory in the elderly (Nissim et al., 2019; Indahlastari et al., 2021), and although they have been used in bilingual language control research showing some promising effects in studies focusing on younger bilinguals (Hämäläinen et al., 2018; Radman et al., 2018; Jost et al., 2020; Tong et al., 2020; Zhu and Sowman, 2020; Vaughn et al., 2021), these methods are virtually unknown in the literature on bilingualism and cognitive aging.

By way of summarizing and concluding, we start here by contextualizing and situating the body of research we have reviewed beyond the science, that is, within the real world, for the benefit of multiple stakeholders for whom this research has impact and implications. After all, understanding what measures one can take to preemptively combat cognitive aging and age-related neuropathology is a societal imperative, not least given the current numbers of older individuals in our societies and the continued projections of increased life expectancy as advances in health improve (Kinsella and He, 2009). There is no question that quality of life issues combine with significant economic implications to make the understanding, predicting, and treating of cognitive aging a universal concern.

Research on drug-based interventions for most age-related neurocognitive pathologies have, to date, produced far-from-satisfying results (Dyer et al., 2018). At present, there is no ground-breaking pharmacological treatment for AD, for example. Unless or until, such treatments become available, reducing risk factors by engaging in preventative measures is the best option for adding years to our healthy (cognitive) life expectancy. If some degree of protection can be afforded through ecological everyday activities, i.e., lifestyle choices, then knowing what these are and how to maximally harness their effects becomes a critical part of dealing with what is sharply becoming a health crisis in our increasingly aging populations worldwide. According to the WHO, more than 50 million people globally are currently affected by dementia (World Health Organization, 2019). This number is likely to be under-representative given the inherent difficulty, cultural and ethnoracial disparities in diagnoses. Even still, the numbers are staggering and the costs are high at multiple levels. Global estimates in 2016 suggested that the total global economic costs of caring for elderly people with dementia then reached nearly a trillion dollars (Xu et al., 2017). To place these numbers in context, Xu et al. (2017) systematic review indicated that by 2017 the average cost per individual case in Europe had risen to just over €32,500. For sake of comparison, the average annual median equivalized income in the EU-27 in 2018 was just under €17,000 (even at the top end of the range, Luxembourg, it was just over €32,500). At present, in the USA alone, costs for dealing with dementia already surpass 300 billion dollars annually with estimates suggesting it will reach one trillion dollars a year as the American population ages (Wong, 2020). These numbers are not comprehensive, of course, relating only to dementia. In other words, when you factor the economic costs associated with non-pathological cognitive aging, MCI and a myriad of other age-related neurodegenerative disorders the encumbrance skyrockets. Clearly, the economic burden of cognitive decline is undeniable and will only continue to grow.

Quality of life costs are much more difficult to gauge. They are also much closer to home, so to speak, as they manifest at the individual level. While difficult to quantify in the first place and compare across individuals, there is little doubt that such costs for elderly individuals themselves, their families and communities are immeasurably high. In these contexts, it becomes socially responsible, beyond scientifically relevant, to research what factors might be within human control to optimize prevention. Studying lifestyle enrichment factors that contribute to successful aging (e.g., via reserve accrual) offers hope that evidence-based recommendations, if not interventions, could be harnessed to ameliorate cognitive aging and/or dementia risk. Factors such as years of education, general mental stimulation, healthy diet, leisure activities, and occupational attainment are positively linked to greater cognitive health (see e.g., Cheng, 2016). And while reserve accrual is typically a happenstance beneficial byproduct of lifestyle enrichment activities, some research suggests that targeted intervention can also be a reserve maintainer/provider. Cognitive training—repetition-based intervention activities that attempt to proactively use scientifically determined environmental enrichment to stretch neuroplasticity—seems to have some effect in boosting cognitive functions in older age (Brum et al., 2009; Belleville et al., 2011; Kane et al., 2017), including foreign language training with seniors (Meltzer et al., 2021).

In this context, bilingualism as a potential contributor to successful aging stands out for several important reasons. As we maintained from the very outset of this article, there is nothing specific to bilingualism *per se* that should privilege it over any other potential successful aging contributor. That said, there are some rather special things that apply to bilingualism—summarized below as the three “-*itie*s”: ubiqu*ity*, equ*ity* and ecological valid*ity—*that do not equally apply to other known factors. Firstly, there is the issue of *ubiquity* on at least two levels. Although this is not so readily appreciated by honing in on the Western world, in particular where English is the (*de facto*) dominant, native language, bi-/multilingualism characterizes the majority of the world’s population (De Houwer, 2021). It is, therefore, naturally occurring at large scale. Bilingualism is also ubiquitous in the sense of the mental processes it evokes in the individual. As mentioned, languages in bilinguals are always maintained with some level of activation, which means that cognitive and language control are constantly engaged to manage the languages and their use. This makes bilingualism temporally unique from other contributors like, for example, physical exercise which is typically much more limited in this respect.

It is reasonable to make an argument for the increased *equity* of bilingualism over the vast majority of other lifestyle factors. Let us consider the listing of potential ameliorative lifestyle factors above to elucidate the point. Having the financial means and/or time for higher degrees of education, healthy diet and physical exercise are all inexorably conditioned by socioeconomic status, often negatively impacting disproportionately racial and ethnic minorities. This is not the case for bilingualism. Some of the most naturally diverse linguistic parts of the world, where languages live in close contact and bilingualism dominates as the societal norm, are among the most economically challenged globally. In so-called monolingual dominant societies, bilingualism is often—not exclusively—most prolific in minority (migrant) populations, which are often socio-economically disadvantaged. Bilingualism need not cost anything, is not a privilege of the few and is often itself an inherent part of minority communities. As a result, it is reasonable to suggest that if engagement with bilingualism turns out to be a *bona fide* lifestyle factor for successful cognitive aging, it is generally more accessible than many other factors. Thus, understanding its connection and nuances with aging and neurodegeneration better, is imperative to effectively and most accurately promote and harness its potential to serve universal health needs in a socially just way. This is especially important as we know that there are ethnoracial disparities for aging, not least, in dementia diagnosis, incidence and treatment (Chin et al., 2011).

Finally, we turn to what we consider the *ecological validity* of bilingualism in this domain. If bilingualism eventually makes the list of recommended activities for longer healthy life expectancy—such as healthy diet and exercise have—then the utility of creating it where it does not naturally exist is likely to expand. Under a reality where new language learning is used as a targeted type of intervention—either in older adults and/or redesigned, prioritized as part of early schooling and continuing education—pointing out co-occurring benefits to doing so is warranted. Learning a new language—at any age but perhaps especially for older adults—as opposed to other types of executive control training has greater real-world applicability. Acquiring new languages can increase individual social networks, encourages new leisure activities (e.g., travel), increases cross-cultural awareness and is transferable to the real world by opening new avenues for communication. Moreover, given that language embodies a large system and learning one requires incremental scaffolding of structure and engages many neurological subsystems, language learning could have keen, immediate and more diffuse effects in older age than bilingual language selection alone has after learning has taken place at younger ages. It might also mean that language learning as a cognitive training intervention is simply more engaging than, for instance, playing Sudoku. If bilingualism as an intervention even only equals the protective potential of other cognitive training (Meltzer et al., 2021), it would be worth promoting for its ecological validity value.

## Author Contributions

All authors listed have made a substantial, direct, and intellectual contribution to the work, and approved it for publication.

## Conflict of Interest

The authors declare that the research was conducted in the absence of any commercial or financial relationships that could be construed as a potential conflict of interest.

## Publisher’s Note

All claims expressed in this article are solely those of the authors and do not necessarily represent those of their affiliated organizations, or those of the publisher, the editors and the reviewers. Any product that may be evaluated in this article, or claim that may be made by its manufacturer, is not guaranteed or endorsed by the publisher.

## Footnotes

1 We do not preclude a dynamic relationship with how measures of quality and quantity of bilingual experience factors will engage with others, for example, age of onset of bilingualism, linguistic proficiency, age at time of testing, etc. For example, recent research from our lab shows that individual differences in bilingual language experience matter independently of when one first became bilingual—as in the case of adults who were simultaneous child bilinguals compared to post-pubescent second language learners—yet age of acquisition matters for how this plays out (Pereira Soares et al., 2021). In other words, bilingual language engagement explains individual differences in brain oscillatory dynamics at rest and during task performance (Flanker) in both sets of bilinguals, but the brain patterns in each are distinct not by age at time of testing, but indeed by age at the time they first became bilingual.
